# Dermal Fibroblasts Modulate Migration and Phenotype of Infiltrating Monocytes in Skin-Derived Extracellular Matrix Hydrogels

**DOI:** 10.3390/gels12040269

**Published:** 2026-03-24

**Authors:** Xue Zhang, Meng Zhang, Linda A. Brouwer, Martin C. Harmsen

**Affiliations:** 1 Department of Pathology and Medical Biology, University Medical Centre Groningen, University of Groningen, Hanzeplein 1 (EA11), 9713 GZ Groningen, The Netherlands; x.zhang02@umcg.nl (X.Z.); zhangmengmed@gmail.com (M.Z.); l.a.brouwer@umcg.nl (L.A.B.); 2 Groningen Research Institute for Asthma and COPD (GRIAC), University Medical Centre Groningen, University of Groningen, Hanzeplein 1 (EA11), 9713 AV Groningen, The Netherlands

**Keywords:** extracellular matrix hydrogel, immune cell infiltration, macrophage-fibroblast crosstalk, matrix remodeling, angiogenesis

## Abstract

Modeling immune cell recruitment within a wound-relevant microenvironment remains challenging. Here, we developed a novel skin-derived extracellular matrix (ECM) hydrogel model to study monocyte (THP-1) entry and phenotypic changes within a dermal fibroblast-populated (NHDF) matrix. The main novelty of this study is that it compares the effects of fibroblast-derived soluble signals and active monocyte infiltration in a 3D biomimetic model. Signaling by fibroblast-secreted soluble factors enhanced a pro-angiogenic secretome (e.g., >3-fold upregulation of VEGFA at day 1) and promoted endothelial tube formation (increasing network junctions to 1.16 ± 0.16 vs. 0.93 ± 0.23 in monoculture). In contrast, this paracrine signaling did not induce the matrix-driven pro-fibrotic response in hydrogels. Crucially, physical immune infiltration restricted monocyte penetration (mean depth of 8.92 ± 2.27 μm vs. 121.1 ± 15.9 μm in monoculture at day 5), reduced hydrogel-induced myofibroblast activation (decreasing α-SMA+ cells from 79.1% to 54.3% upon initial contact), and was associated with slower collagen loss during the early phase. (retaining a high-density collagen ratio of 3.46 ± 0.33 vs. 2.02 ± 0.29 in monoculture at day 1). These observations were accompanied by a shift toward a matrix-stabilizing profile, including increased TIMP expression and reduced pro-fibrotic markers. (ACTA2 and COL1A1). By including active immune infiltration (which was absent in previous tSVF models), we capture the transition from inflammation to the proliferation stage. Although the later stages of extensive ECM remodeling appear suppressed here, they may occur as repair progresses. Overall, our findings highlight that the immune cell is a key regulatory component for coordinating matrix preservation and vascular support. Importantly, this model replicates the early phases of wound healing, a stage where the monocyte–fibroblast secretome supports endothelial network formation. We established this innovative 3D ECM hydrogel system as a practical and physiologically relevant platform to investigate immune–matrix–stromal crosstalk.

## 1. Introduction

**Figure 1:**
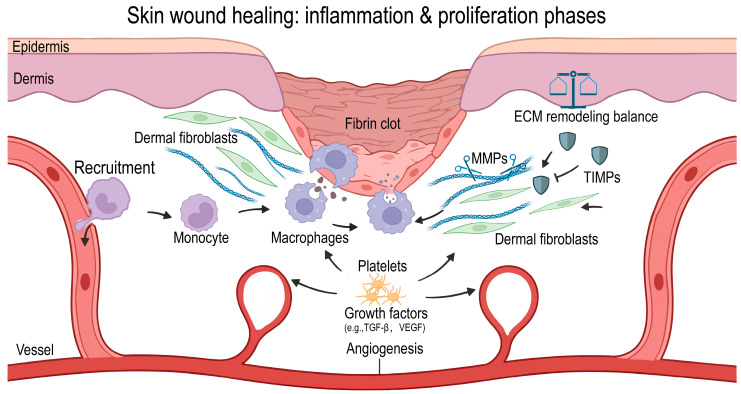
Schematic representation of the inflammatory and proliferative phases of cutaneous wound healing. After tissue damage and hemostasis (fibrin clot formation), wound repair is initiated by activating circulating monocytes from the vasculature into the wound bed (**Left**). Monocytes differentiate into macrophages and secrete key growth factors (e.g., TGF-, VEGF), which stimulate angiogenesis and fibroblast activation (**Center**). As the wound healing area grows in the proliferative stage, resident fibroblasts synthesize and remodel ECM by regulating the enzymatic balance between matrix metalloproteinases (MMPs) and tissue inhibitors of metalloproteinases (TIMPs) (**Right**). Crosstalk between immune cells and stromal niche determines the balance between effective tissue repair and scarring. Created with BioRender.com.

Following injury, the skin, as the body’s outermost barrier organ, initiates wound healing through stage-specific processes, including hemostasis, inflammation, proliferation, and remodeling [1,2]. This progression relies on tightly regulated mutual interactions between immune cells and dermal fibroblasts, which links primary inflammation to subsequent tissue repair [3,4]. Early in the inflammatory phase, circulating neutrophils and monocytes infiltrate the wound, clearing pathogens and necrotic tissue; later, macrophages secrete cytokines and growth factors that support wound healing. To pave the way for subsequent phases, a vascular network is established starting in the early phase (Figure 1 [5]). The subsequent phases, proliferation and remodeling, involve dermal fibroblast migration, proliferation, and the synthesis of ECM, enabling granulation tissue formation, collagen restructuring, and the ultimate formation of a scar [6]. Finally, wound healing resolves or in case of persistent triggers, may ensue in chronic inflammation. In fact, disruption at any stage can result in nonhealing wounds, excessive scarring, or fibrosis [7].

The dynamic process of wound healing involves multiple cell types and extracellular matrix (ECM) remodeling across overlapping stages. This complexity requires advanced in vitro models to accurately recapitulate cell–cell and cell–ECM interactions. Conventional 2D models, i.e., on stiff tissue culture plastic, compromise the mechanical properties of the relatively soft skin and are therefore inadequate to date [8]. Such conditions markedly alter cell morphology and mechanosensing, driving cells into an activated state that deviates from physiological behavior [9]. Crucially, in vivo tissues, including skin, are organized in three dimensions: cells are embedded or intercalated within an ECM scaffold and continuously sense and remodel their surroundings through adhesion, tension, and traction. Two-dimensional culture cannot reproduce the migratory routes and spatial distributions of cells within a 3D matrix, nor can it mimic cellular responses to local microenvironmental gradients such as nutrients, oxygen, and signaling molecules [10].

ECM represents a highly ordered and dynamically tuneable biomaterial, composed primarily of fibrillar proteins (such as type I and III collagens and elastic fibers) and a proteoglycan-rich polysaccharide gel containing sulfated glycosaminoglycans [11]. With advances in decellularization and tissue-engineering techniques, the preparation of ECM hydrogels from decellularized tissues—where cellular components are removed while the ECM structure and composition are preserved—has become an increasingly important strategy for constructing three-dimensional in vitro models [12]. Skin-derived ECM hydrogels largely retain the dermis-specific collagen network, basement membrane constituents, and a variety of embedded bioactive factors [13,14,15]. Therefore skin-derived ECM hydrogels represents a suitable biomaterial scaffold that recapitulates multiple key biochemical and mechanical properties of native skin extracellular matrix in vivo [16].

Angiogenesis is a key determinant of cutaneous wound healing and is tightly regulated by the crosstalk between immune cells and fibroblasts [17,18]. Both cell types secrete pro-angiogenic factors, including vascular endothelial growth factor (VEGF) and fibroblast growth factor-2 (FGF2), which promote endothelial cell migration and proliferation [19]. Newly deposited ECM produced by fibroblasts, such as collagens and glycosaminoglycans (GAGs), has been shown to support endothelial sprouting and lumen formation [16]. In addition, the intrinsic physical and biochemical properties of the ECM regulate vascular network formation and influence fibroblast-based matrix remodeling [20]. In a clinical trial, we previously observed that performing two sequential fat grafting procedures in mature cutaneous scars—at baseline and 3 months later—induces sustained macrophage infiltration, accompanied by progressive blood vessel ingrowth [21]. In contrast, in an in vitro culture system consisting of skin ECM-derived hydrogels containing a major component of fat, i.e., tissue-derived stromal vascular fraction (tSVF), vascular network formation did not occur [22]. This discrepancy suggests that, under these in vitro conditions, the lack of continuous recruitment of circulating monocytes/macrophages may hinder angiogenesis. These observations emphasize the need to develop a three-dimensional skin ECM model that concurrently incorporates immune cells, fibroblasts, and dynamic matrix remodelling to systematically evaluate their roles in wound repair. Previous studies typically examined these aspects separately from immunological, fibrotic, or materials science perspectives. Rarely has a single system simultaneously tracked the temporal evolution of matrix composition, fibrotic markers, and pro-angiogenic potential. To address this gap, we integrated the THP-1 monocytic cell line and normal primary human dermal fibroblasts (NHDFs) into 3D skin ECM hydrogels. The novelty of our work is that the dynamic 3D biomimetic platform distinguishes soluble paracrine signals from physical monocyte infiltration in native-like skin ECM scaffold. In this model, THP-1 cells recapture monocytic immune cell recruitment, whereas matrix-remodeling fibroblasts are used. Unlike traditional in vitro models that rely on static co-cultures, homogeneous 3D mixtures, or stiff 2D substrates, our new system actively simulates the spatial migration of innate immune cells from the circulation into the wound bed. We hypothesized that, within a skin-derived ECM environment, distinct patterns of immune cell–fibroblast interactions give rise to divergent outcomes in inflammation, fibrosis, ECM remodelling, and pro-angiogenic capacity. By performing time-resolved sampling and applying multiplex readouts, this model enables a systematic dissection of the regulatory role of the immune–fibroblast–ECM network in cutaneous wound healing.

## 2. Results and Discussion

### 2.1. Results

#### 2.1.1. Direct Co-Culture with NHDFs Restricts THP1 Infiltration in Skin-Derived Hydrogels

**Figure 2:**
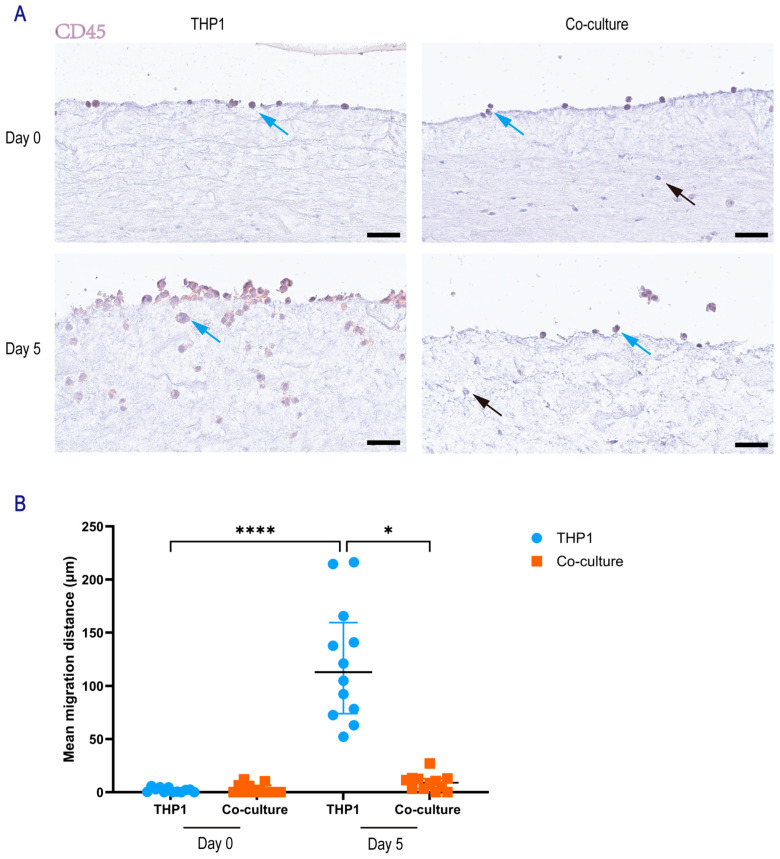
Migration of THP-1 cells in the direct co-culture system. (**A**) Representative CD45 immunohistochemistry (IHC) images showing THP-1 migration in skin-derived hydrogels in the THP-1 monoculture group and the direct co-culture group at day 0 and day 5. THP-1 cells are CD45-positive (blue arrows), whereas NHDF are CD45-negative (black arrows). Scale bar, 50 μm. (**B**) Quantification of THP-1 migration depth within the hydrogels (Day 0 THP-1, Day 0 co-culture, Day 5 THP-1, and Day 5 co-culture) using Fiji (version 1.53t). For each image, the five THP-1 cells located farthest from the hydrogel surface were identified and the perpendicular distance from each cell to the hydrogel surface was measured; the mean of these five distances was used as the migration metric for that image. Data was from four independent experiments; for each sample, three random regions were analyzed. Each dot represents one random region. Data was presented as mean ± SEM. Statistical significance was assessed by one-way ANOVA with Tukey’s multiple-comparisons test. Significance was indicated as * *p* < 0.05, and **** *p* < 0.0001.

To investigate the migratory behavior of THP-1 monocytes in the presence of dermal fibroblasts, we utilized a skin-derived hydrogel model comparing THP-1 monocultures to direct co-cultures with NHDFs. Representative immunohistochemistry (IHC) images revealed distinct cellular distributions within the hydrogels (Figure 2A). THP-1 cells, identified by CD45 expression (CD45+), were distinguishable from the CD45-negative NHDFs. Qualitatively, THP-1 cells in the monoculture group appeared to migrate deeply into the hydrogel by day 5, whereas THP-1 migration appeared markedly restricted in the co-culture group, with cells remaining closer to the hydrogel surface.

Quantitative analysis of migration depth confirmed these observations (Figure 2B). At day 0, baseline migration depths were negligible and comparable between the monoculture (1.95 ± 0.63 μm) and co-culture groups (2.96 ± 1.34 μm; *p* > 0.99). By day 5, THP-1 cells in the monoculture group exhibited a substantial increase in migration depth, reaching a mean depth of 121.1 ± 15.9 μm, which was higher than the day 0 baseline (*p* < 0.0001). In contrast, the presence of NHDFs in the co-culture group inhibited THP-1 migration at day 5 (8.92 ± 2.27 μm) compared to the day 5 monoculture group (*p* < 0.0001). Furthermore, the migration depth in the day 5 co-culture group did not differ from the day 0 baseline (*p* > 0.05), suggesting that the presence of fibroblasts prevented THP-1 invasion into the hydrogel over the 5-day period.

#### 2.1.2. Influence of THP-1 Cells on NHDFs Phenotype

**Figure 3:**
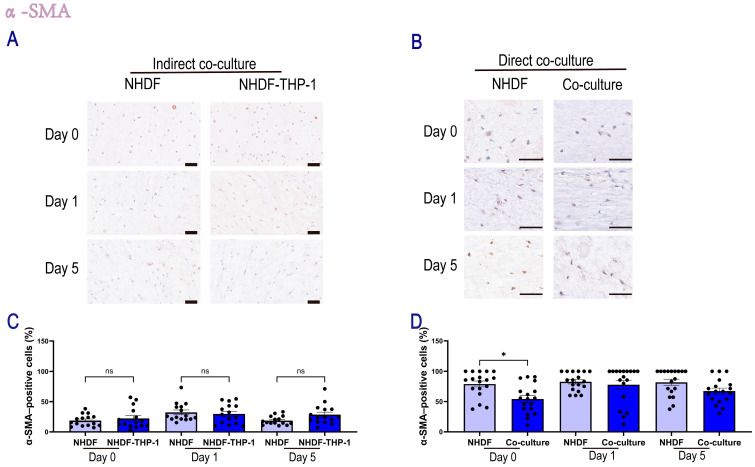
Quantification of α-SMA-positive NHDFs in the direct and indirect co-culture system. Representative DAB-based IHC images showing α-SMA expression in NHDFs, where positive staining appeared brown and negative cells displayed only blue hematoxylin-counterstained nuclei. (**A**) Indirect co-culture: α-SMA staining of NHDFs at day 0, day 1, and day 5. Scale bar, 100 μm. (**B**) Direct co-culture: α-SMA staining of NHDFs at day 0, day 1, and day 5. Scale bar, 50 μm. (**C**) Quantification of α-SMA–positive NHDFs in the indirect co-culture system at day 0, day 1, and day 5. (**D**) Quantification of α-SMA–positive NHDFs in the direct co-culture system at day 0, day 1, and day 5. The percentage of α-SMA–positive NHDFs was calculated in Fiji (version 1.53t) as (number of DAB-positive NHDFs/total NHDFs) × 100 for each field. Data were obtained from *n* = 6 independent samples, with three random regions per sample analyzed. Each dot represents one randomly selected region measurement. Data are presented as mean ± SEM. Statistical significance was assessed by one-way ANOVA with Tukey’s multiple-comparisons test. Significance was indicated as * *p* < 0.05, while “ns” indicates no significant difference.

To investigate the phenotypic state of fibroblasts, we assessed the expression of α-SMA, a hallmark marker of (myo)fibroblast activation, using immunohistochemical analysis. To evaluate whether soluble factors secreted by monocytes regulate fibroblast activation, we analyzed NHDFs in an indirect co-culture system (Figure 3A,C). The percentage of α-SMA-positive cells in this setup remained low and comparable to that in the monoculture controls throughout the 5-day period. For instance, at day 5, the α-SMA positive rate in the indirect co-culture group (28.6 ± 4.2%) was not different from the NHDF monoculture group (18.8 ± 1.9%; *p* > 0.05). These results suggest that paracrine signaling alone was insufficient to alter the α-SMA expression profile of NHDFs in this model. In contrast, direct co-culture with monocytes suppressed fibroblast activation (Figure 3B,D). Unlike the indirect system, direct co-culture suppressed the high baseline activation of NHDF controls observed in the hydrogels. At day 0, the presence of THP-1 cells reduced the percentage of α-SMA-positive fibroblasts from 79.1 ± 4.9% (THP-1) to 54.3 ± 5.7% (Co-culture; *p* < 0.05). A similar trend of reduced activation was maintained at day 5 (67.3 ± 4.8% vs. 81.9 ± 5.2%), although this difference did not reach statistical significance (*p* > 0.05). Collectively, these findings indicated that cells in contact with a shared ECM communicate and that locally present monocytes attenuated the hydrogel-induced myofibroblast activation.

#### 2.1.3. Indirect Co-Culture Induces Rapid Collagen Degradation

**Figure 4:**
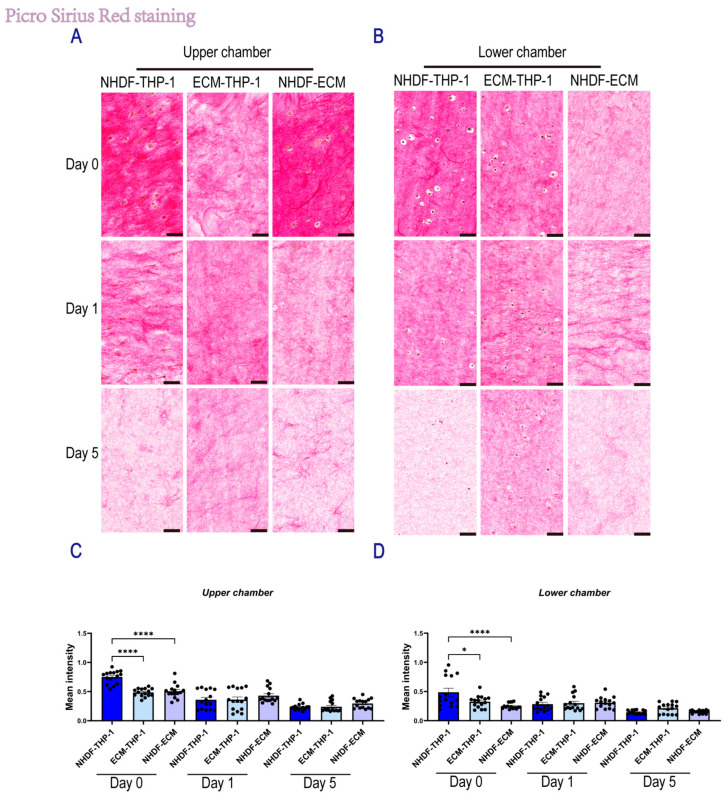
Picrosirius Red staining of skin-derived ECM hydrogel under indirect co-culture conditions. (**A**) Representative PSR staining images of skin-derived ECM hydrogels in the upper Transwell insert under the indirect co-culture system at Day 0, Day 1, and Day 5 (scale bar, 50 μm). (**B**) Representative PSR staining images of skin-derived ECM hydrogels in the lower Transwell compartment under the indirect co-culture system at Day 0, Day 1, and Day 5 (scale bar, 50 μm). (**C**,**D**) Quantification of the mean intensity of collagen fibers. Mean intensity was measured in Fiji by defining regions of interest (ROIs) and recording the Mean Gray Value. Background intensity was obtained from an adjacent unstained area and subtracted from each ROI measurement to yield the background-corrected mean intensity for each field. For the indirect co-culture system, *n* = 3 samples, with five random fields quantified per sample; each dot represents a random-field measurement. Data are presented as mean ± SEM. Statistical significance was assessed using two-way ANOVA with Tukey’s multiple comparisons test. * *p* < 0.05, and **** *p* < 0.0001.

**Figure 5:**
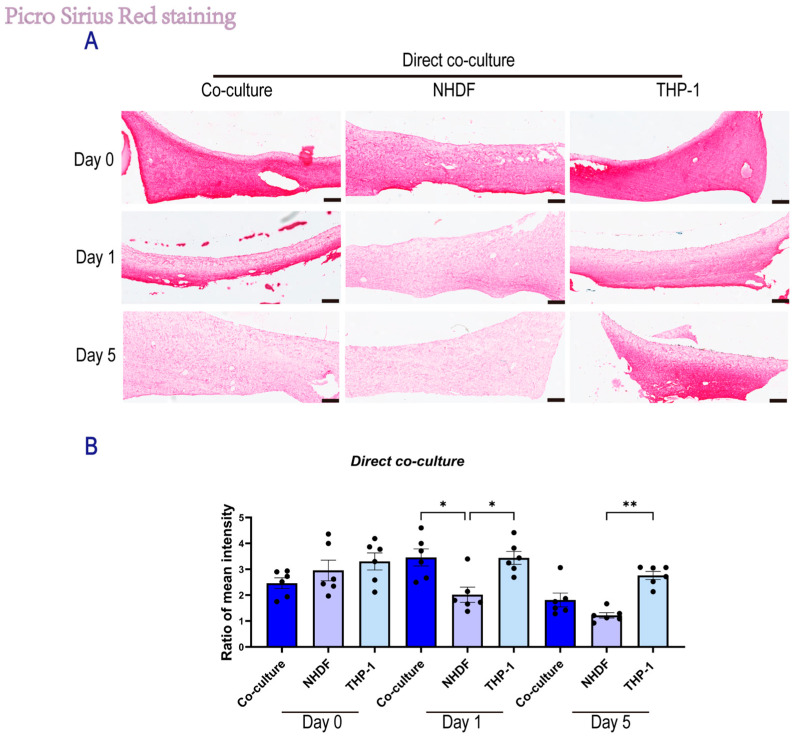
PSR staining of skin-derived ECM hydrogel under direct co-culture conditions. (**A**) Representative PSR staining images of skin-derived ECM hydrogels under the direct co-culture system at Day 0, Day 1, and Day 5 (scale bar, 250 μm). (**B**) Quantification of the mean intensity ratio of collagen fibers in the direct co-culture system. For each sample, three random fields were acquired from the upper low-density region and three random fields from the lower high-density region. Background-corrected mean intensity was calculated for each field (six ROIs total), and the intensity ratio was computed as the mean intensity of the lower region divided by the mean intensity of the upper region. In the direct coculture system, *n* = 6 samples, with one random field quantified per sample; each dot represents a random field measurement. Data are presented as mean ± SEM. Statistical significance was assessed using two-way ANOVA with Tukey’s multiple comparisons test. * *p* < 0.05, ** *p* < 0.01.

To determine the impact of interactions between monocytes (THP-1) and dermal fibroblasts (NHDF) on matrix remodeling, we assessed collagen fiber density using picrosirius red (PSR) staining in both indirect and direct co-culture systems over 5 days (Figure 4 and Figure 5).

In the indirect co-culture system, collagen remodeling dynamics were statistically analyzed using a two-way ANOVA to evaluate the interaction between culture time and cell composition. In the upper transwell insert containing NHDFs (Figure 4A,C), these analyses showed a direct correlation between time and culture condition (*p* < 0.0001), indicating distinct remodeling kinetics in different groups. Specifically, at day 0, the NHDF-THP-1 group exhibited a significantly higher collagen density (0.75 ± 0.03) compared to the NHDF-ECM control (0.51 ± 0.03) and ECM-THP1 group (0.49 ± 0.02, *p* < 0.001). However, this initial state was followed by a rapid and substantial degradation phase. By day 5, collagen intensity in the NHDF-THP-1 group had precipitously declined to 0.23 ± 0.01, reaching a level statistically comparable to the NHDF-ECM control (0.30 ± 0.02, *p* > 0.05). Notably, although all groups showed a significant time-dependent reduction in collagen intensity (*p* < 0.0001 for the main effect of time), the NHDF-THP-1 group showed the steepest decline in slope. This suggests that paracrine signaling from monocytes accelerates fibroblast-mediated matrix degradation, driving the system from a high-density state to a degraded state more rapidly than in monoculture. In the lower chamber containing THP-1 cells (Figure 4B,D), a similar trend was observed. Statistical analysis at day 0 revealed that the NHDF-THP1 group (0.49 ± 0.07) possessed a significantly higher baseline collagen density compared to the NHDF-ECM control (0.25 ± 0.01, *p* < 0.01) and ECM-THP1 group (0.33 ± 0.03, *p* < 0.05). This suggests that the co-culture environment induces immediate matrix compaction in both chambers. However, similar to the upper chamber, this difference was transient. By day 5, collagen intensity in the NHDF-THP-1 group decreased to 0.15 ± 0.01, becoming indistinguishable from the NHDF-ECM control (0.15 ± 0.01, *p* > 0.05). These findings confirm that the accelerated remodeling dynamics—characterized by an initial high-density state followed by rapid degradation—are a consistent feature of the co-culture system in both cellular compartments.

#### 2.1.4. Direct Co-Culture Attenuates Early Fibroblast-Mediated Matrix Degradation

In the direct co-culture system, we utilized the ratio of collagen intensity between the lower (NHDF-embedded) and upper (THP-1 on top) regions of the cross-sectioned hydrogels to quantify fibroblast-mediated matrix degradation (Figure 5). A ratio > 1 indicates that collagen density in the fibroblast layer remains higher than in the bulk matrix, i.e., indicating preservation. In contrast, a decrease in ratio reflects active proteolytic degradation within the fibroblast microenvironment. Two-way ANOVA revealed a strong correlation between time and culture condition (*p* < 0.05), which indicates a distinct temporal regulation of proteolytic activity. Notably, analyses at day 1 revealed that monocytes inhibited the matrix degradation by fibroblasts. In the NHDF monoculture group, the ratio dropped rapidly from day 0 to day 1 (2.02 ± 0.29), indicating that in the absence of monocytes, fibroblasts immediately initiated matrix degradation. In contrast, the co-culture group maintained a significantly higher ratio at day 1 (3.46 ± 0.33, *p* < 0.05 vs. NHDF), which was comparable to the initial state. This retention of a high density ratio suggests that the physical presence or matrix-dependent signaling by THP-1 cells at least transiently suppressed collagen-degradation by NHDFs during the early culture phase. However, this inhibitory effect appeared to be transient as by day 5, the ratio in the co-culture group had declined to 1.81 ± 0.27, approaching that of the NHDF monoculture group (1.22 ± 0.10; *p* > 0.05). This finding might replicate the transition from early wound healing to the next phase, i.e., from angiogenesis to matrix remodeling. Collectively, these findings indicate that THP-1 cells exert an early protective influence, preventing the rapid matrix degradation typically driven by fibroblasts.

#### 2.1.5. Secretome from NHDF/THP-1 Co-Cultures Promotes In Vitro Angiogenesis

**Figure 6:**
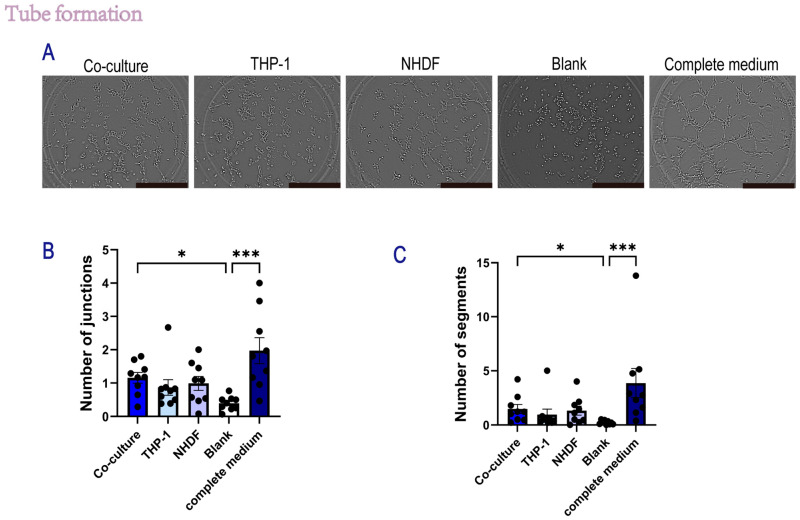
Conditioned media from an indirect co-culture system modulate HUVEC tube formation on Matrigel. Human umbilical vein endothelial cells (HUVECs) were seeded on Matrigel, and tube formation was evaluated by bright-field microscopy. HUVECs were stimulated with 24 h conditioned media (CM) collected from an indirect Transwell co-culture system: (1) experimental co-culture CM (NHDF in the upper chamber + THP-1 in the lower chamber), (2) THP-1 monoculture CM (THP-1 in the lower compartment only), (3) NHDF monoculture CM (NHDF in the upper insert only), (4) basal medium (RPMI 1640), and (5) HUVEC complete growth medium. Representative bright-field images were acquired 2 h after seeding. Scale bar: 50 μm. (**A**) Representative bright-field images of HUVEC tube-like structures under the indicated conditions. (**B**,**C**) Quantification of tube formation, including the number of junctions and the number of segments, using identical analysis settings across groups. *n* = 3 independent experiments; each experiment included three technical replicates per condition. For each replicate, one whole-well bright-field image was captured and analyzed. Each dot represents one well. Data are presented as mean ± SEM. Statistical significance was assessed using one-way ANOVA with Tukey’s multiple comparisons test. Horizontal lines with asterisks indicate statistically significant differences between the specific groups connected (* *p* < 0.05, *** *p* < 0.001).

To investigate the angiogenic potential of paracrine factors secreted by the indirect coculture system, HUVECs were seeded on Matrigel and treated with conditioned media (CM) from the indirect co-culture of NHDFs and THP-1 cells, as well as from their respective monocultures. Tube formation was assessed at 2 h post-seeding. As shown in Figure 6A, HUVECs treated with basal medium (Blank) failed to form organized networks, whereas cells treated with complete medium (positive control) formed robust, interconnected tube-like structures.

Quantitative analysis of topological parameters revealed that co-culture CM significantly increased the complexity of the endothelial network compared with the basal medium (free of growth factors) control. Specifically, secretome from the co-culture group showed a marked increase in both the number of junctions (*p* < 0.05 vs. Blank, Figure 6B) and the number of segments (*p* < 0.05 vs. Blank, Figure 6C). The mean number of junctions in the co-culture group was approximately 1.16 ± 0.16 (mean ± SEM), which was higher than that of the THP-1 monoculture group (0.87 ± 0.25) and the NHDF monoculture group (0.93 ± 0.23). A similar trend was observed in the segmentation analysis, in which the co-culture CM induced a higher number of vessel segments (1.57 ± 0.39) than the THP-1 (0.94 ± 0.51) and NHDF (1.30 ± 0.43) monocultures. These results suggest that the secretome generated by the interaction between NHDFs and THP-1 cells exhibits pro-angiogenic properties that modulate angiogenesis in vitro.

#### 2.1.6. Paracrine Signaling in Indirect Coculture Modulates the Gene Expression Profile of THP-1

**Figure 7:**
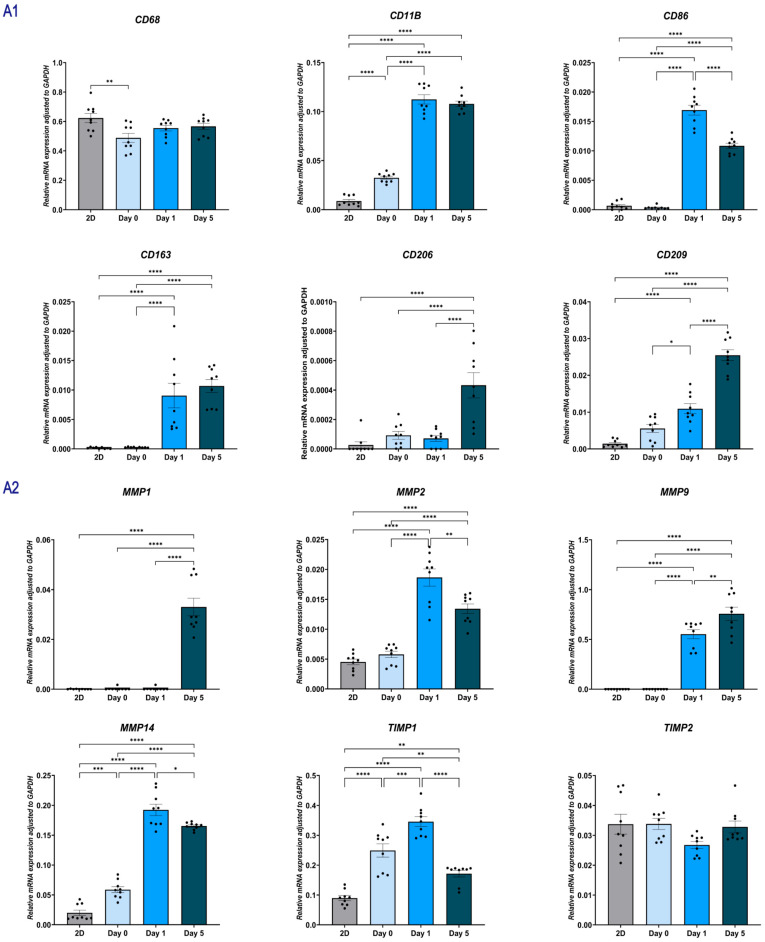

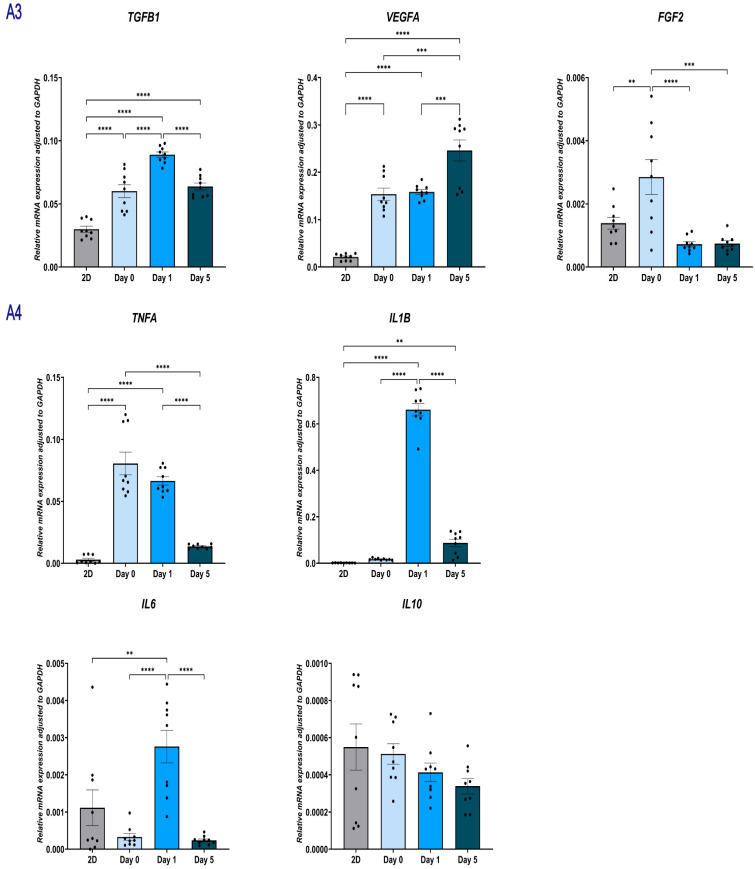

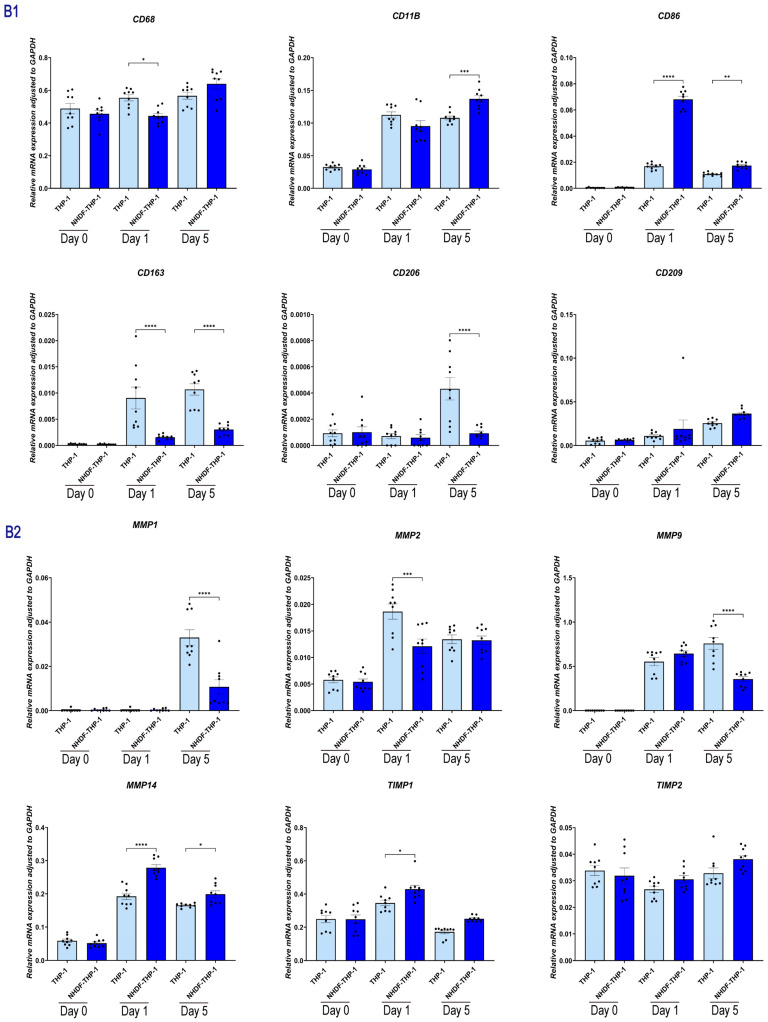

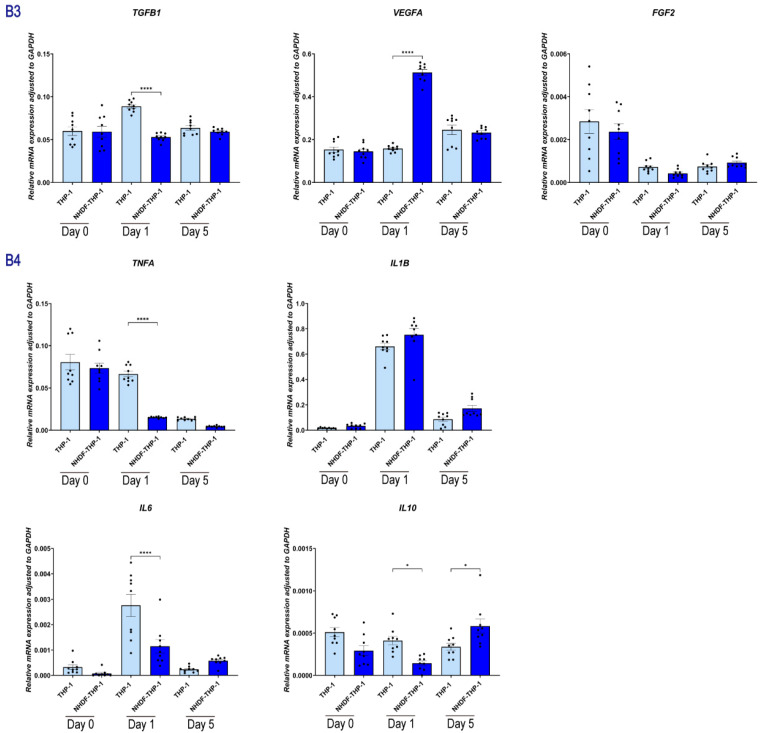
Gene expression profiles of THP-1 cells in an indirect co-culture system. Relative mRNA expression levels were determined by RT-qPCR at Day 0, Day 1, and Day 5. Data were normalized to the internal control *GAPDH* using the Δ Ct method. (**A1**–**A4**) Gene expression in THP-1 cells cultured alone in skin-derived hydrogels (indirect coculture system, group A). Panels show mRNA levels of macrophage surface markers (**A1**), matrix metalloproteinases (*MMPs*) and *TIMPs* (A2), growth factors (**A3**), and inflammatory cytokines (**A4**). (**B1**–**B4**) Gene expression in THP-1 cells co-cultured with NHDF (indirect coculture system, group B). Panels display the same target genes as Group A to assess the paracrine effects of NHDF on THP-1. Results are representative of three independent experiments, with each experiment performed in three technical replicates. Each data point represents a single technical replicate. Statistical significance was analyzed using one-way ANOVA with Tukey’s multiple comparisons test. * *p* < 0.05, ** *p* < 0.01, *** *p* < 0.001, and **** *p* < 0.0001.

To evaluate the influence of NHDFs on macrophage polarization and function, we analyzed the temporal gene expression of THP-1 cells cultured alone (THP-1, Figure 7A) versus those co-cultured with NHDFs (NHDF-THP-1, Figure 7B).

THP-1 macrophage-associated markers and cytokines: Compared with THP-1 monoculture, THP-1 cells in indirect co-culture showed increased expression of *CD68* and *CD11B*. In the same comparison, *CD206* and *CD163* were reduced (*p* < 0.01), whereas *CD209* and *CD86* were increased. Over time, *TNFA* and *IL1B* decreased, whereas *IL6* and *IL10* remained stable or increased (day 5, *p* < 0.05).

Pro-inflammatory Cytokines: The expression of *TNFA* and *IL1B* dropped rapidly in the NHDF-THP-1 group. By Day 5, *TNFA* levels in the NHDF-THP-1 group were markedly lower than in the THP1 monoculture group (Mean ~0.004 vs. ~0.015), indicating a suppression of pro-inflammatory signaling.

Anti-inflammatory cytokines: The expression of the key anti-inflammatory mediator *IL10* was upregulated in the NHDF-THP-1 group by Day 5. *IL6* expression was sustained.

Angiogenic factors: The NHDF-THP-1 group exhibited a rapid upregulation of *VEGFA* specifically at Day 1. The expression levels were over 3-fold higher than in the monoculture group (*p* < 0.0001). By Day 5, *VEGFA* levels returned to baseline, comparable to the monoculture group. *FGF2* expression did not increase in the NHDF-THP-1 system. In fact, it was significantly downregulated at Day 1 compared to monocultures (*p* < 0.01) and showed no significant difference by Day 5. *TGFB1* expression increased in THP-1 monocultures, but it was significantly downregulated in NHDF-THP-1 group at Day 1 (*p* < 0.001). By Day 5, *TGFB1* levels in NHDF-THP-1 group were comparable to those in the monoculture group (*p* > 0.05).

ECM-related genes: The co-cultured THP-1 maintained expression of *MMP9* and *MMP1*. The expression of the inhibitor *TIMP1* was higher in NHDF-THP-1 group at Day 1 compared to monocultures (*p* < 0.05). *TIMP2* showed sustained expression. The expression of *MMP2* was significantly downregulated in the NHDF-THP-1 group during the early phase (Day 1, *p* < 0.01) and showed no significant difference by Day 5. The expression of the membrane-bound *MMP14* was consistently and significantly upregulated in the NHDF-THP-1 group at both Day 1 (*p* < 0.00001) and Day 5 (*p* < 0.01) compared to monocultures.

#### 2.1.7. Paracrine Signaling in Indirect Coculture Modulates the Gene Expression Profile of NHDF

**Figure 8:**
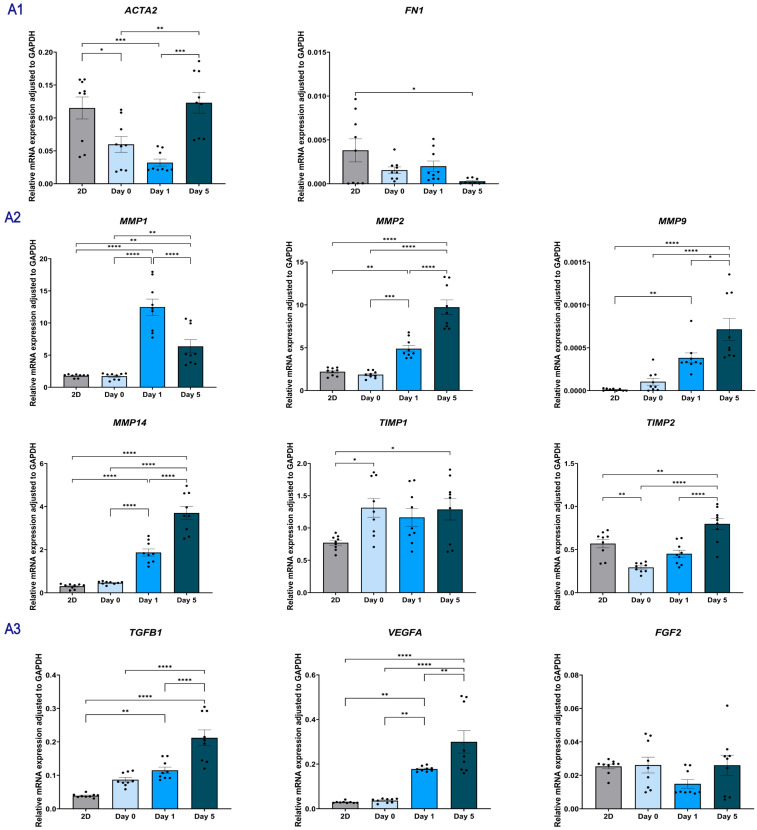

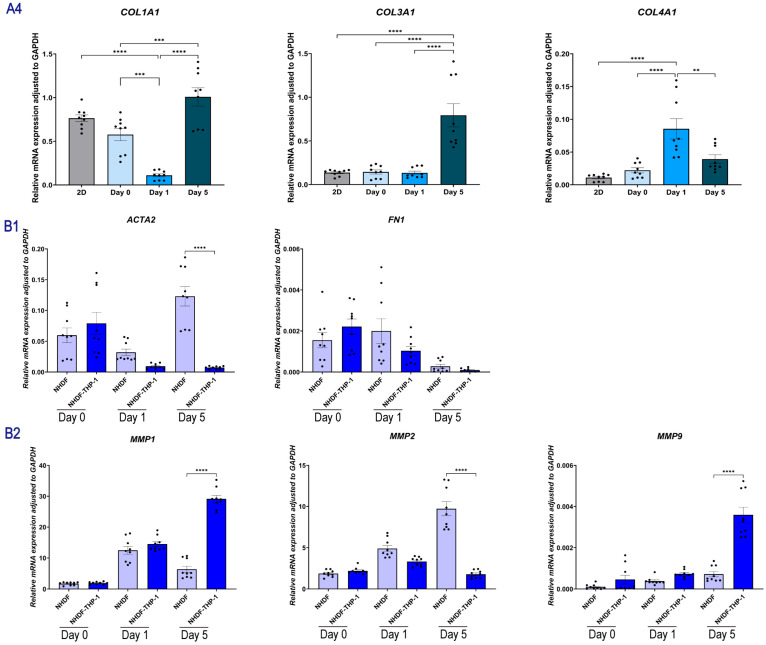

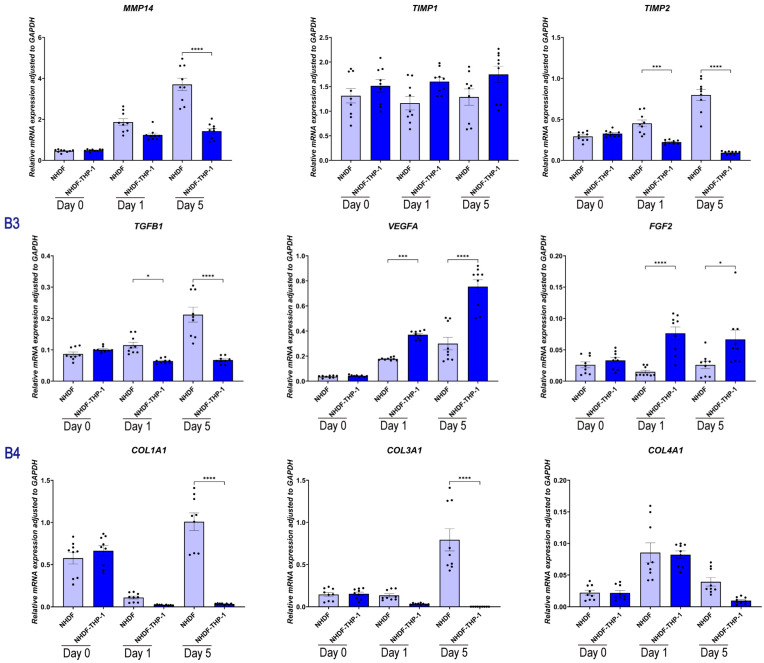
Gene expression profiles of NHDF cells in an indirect co-culture system. (**A1**–**A4**) Gene expression in NHDFs encapsulated in skin-derived hydrogels cultured alone (indirect coculture system, group A). Panels show mRNA levels of fibroblast activation markers (**A1**), *MMPs* and *TIMPs* (**A2**), growth factors (**A3**), and collagen subtypes (**A4**). (**B1**–**B4**) Gene expression in NHDFs co-cultured with THP-1 (indirect coculture system, group B). Panels display the same target genes as group A to evaluate the paracrine influence of THP-1 on fibroblast phenotype and matrix remodeling. Data are presented as mean ± SEM. Results are representative of three independent experiments, with each experiment performed in three technical replicates. Each data point represents a single technical replicate. Statistical significance was analyzed using one-way ANOVA with Tukey’s multiple comparisons test. * *p* < 0.05, ** *p* < 0.01, *** *p* < 0.001, and **** *p* < 0.0001.

To elucidate the impact of macrophage-derived paracrine signals on fibroblast behavior, we performed a comprehensive temporal gene expression analysis of NHDFs cultured alone (Monoculture, Figure 8A) versus those co-cultured with THP-1 cells (NHDF-THP-1, Figure 8B).

Fibroblast activation markers: In the monoculture group, *ACTA2* levels initially dipped at Day 1 (Mean ~0.03) but surged significantly by Day 5 (*p* < 0.001 vs. Day 1), indicating a delayed but potent spontaneous activation. In contrast, the NHDF-THP-1 group maintained a continuous downward trend, resulting in near-complete suppression by Day 5 (*p* < 0.001 vs. monoculture). *FN1* expression remained stable and showed no significant difference between the monoculture and NHDF-THP-1 groups (*p* > 0.05).

ECM-related genes: Co-cultured cells massively upregulated the soluble interstitial *MMP1* (*p* < 0.001) and *MMP9* (*p* < 0.001). Monoculture NHDFs highly upregulated the membrane-bound *MMP14* and *MMP2* by Day 5, with *MMP14* levels reaching a peak of ~3.70. The Co-culture system significantly suppressed this pathway, reducing *MMP14* to ~1.42 (*p* < 0.001) and significantly lowering *MMP2* levels (*p* < 0.001).

Angiogenic factors: Co-cultured fibroblasts expressed *VEGFA* at markedly higher levels than monoculture cells at Day 5 (*p* < 0.001), representing a >2.5-fold amplification.

Similarly, *FGF2* expression was significantly boosted in the NHDF-THP-1 group compared to the monoculture baseline at both Day 1 (*p* < 0.001) and Day 5 (*p* < 0.05). *TGFB1* in monoculture showed a consistent and progressive upregulation from Day 0 to Day 5 (0.08 → 0.21). The Co-culture system significantly blunted this trajectory at all time points (*p* < 0.001).

Collagens: In monoculture, the expression of *COL1A1* dropped sharply at Day 1 but staged a massive rebound by Day 5 (Mean ~1.01), reflecting the onset of aggressive scar deposition. The Co-culture system completely blocked this rebound, keeping *COL1A1* and *COL3A1* levels suppressed at Day 5 (*p* < 0.001). *COL4A1* followed a similar pattern, being significantly downregulated in the Co-culture group by Day 5 compared to the monoculture (*p* < 0.001).

#### 2.1.8. Effects of Direct Co-Culture on Gene Expression of NHDFs and THP-1s

**Figure 9:**
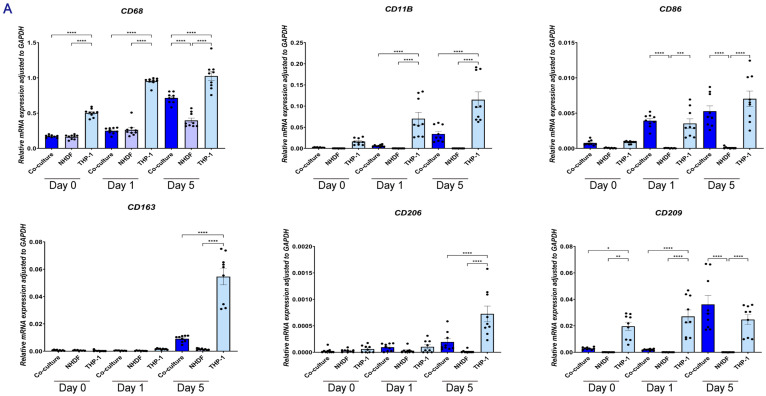

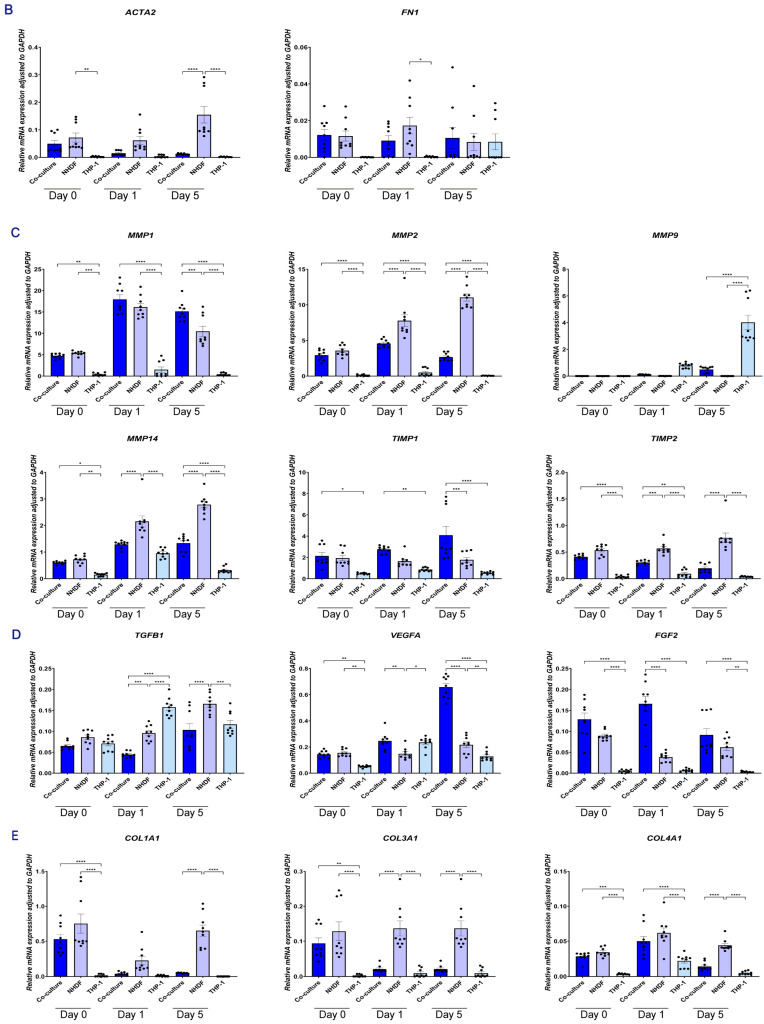

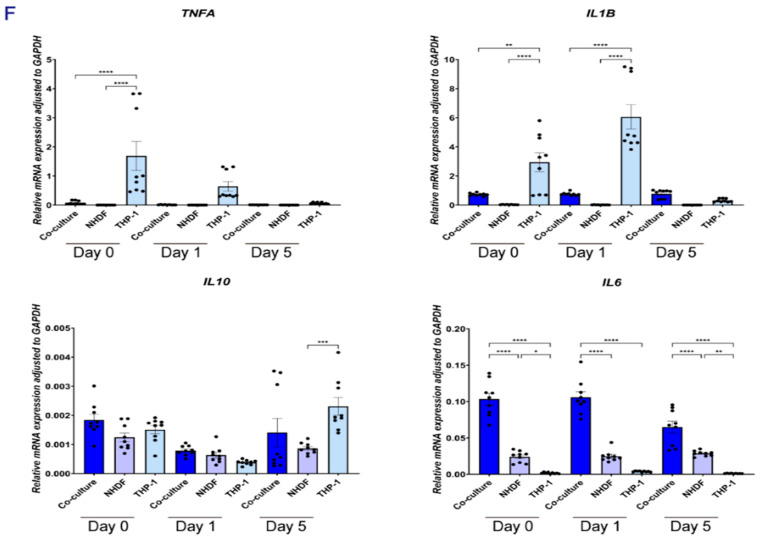
Gene expression profiles of NHDF and THP-1 cells in a direct co-culture system. (**A**) macrophage markers; (**B**) fibroblast activation markers; (**C**) *MMPs* and *TIMPs*; (**D**) growth factors; (**E**) collagens; (**F**) inflammatory cytokines. Data are presented as mean ± SEM. Results are representative of three independent experiments, with each experiment performed in three technical replicates. Each data point represents a single technical replicate. Statistical significance was analyzed using one-way ANOVA with Tukey’s multiple comparisons test. * *p* < 0.05, ** *p* < 0.01, *** *p* < 0.001, and **** *p* < 0.0001.

THP-1 macrophage marker expression (Figure 9A): Compared with THP-1 monoculture, THP-1 cells in direct co-culture showed reduced expression of *CD68*, *CD11B*, *CD163*, and *CD206* (*p* < 0.01). *CD209* increased over time in the direct co-culture group from day 1 to day 5 (*p* < 0.001), whereas this time-dependent increase was not observed in THP-1 monoculture. *CD86* also increased over time.

Angiogenic factors and cytokines (Figure 9D,F): In the direct co-culture group, total *VEGFA* and *FGF2* expression were increased relative to monocultures. *TNFA* and *IL1B* were lower in direct co-culture than in THP-1 monoculture, while *IL6* remained detectable and *IL10* increased.

Selective phenotypic modulation and controlled matrix remodeling: direct physical contact between THP-1 and NHDFs induced a marked, yet selective, shift in the fibroblast ECM program. Compared with NHDF monoculture, the canonical profibrotic markers *ACTA2* (Figure 9B), *COL1A1*, and *COL3A1* (Figure 9E) were strongly downregulated under direct co-culture conditions (*p* < 0.001), indicating a pronounced suppression of the *α-SMA*–associated and fibrillar collagen gene signature (Figure 9E). In contrast, *COL4A1* (Figure 9E) and *FN1* (Figure 9B) were comparatively preserved and, by day 5, returned to levels comparable to the monoculture control. Consistent with these transcriptional changes, analysis at day 1 showed that THP-1 cells exerted a strong inhibitory effect on NHDF-driven ECM degradation (Figure 9C). In parallel, *MMP1* and *MMP9* remained detectable, whereas *MMP14* displayed a relatively low net expression level. Notably, *TIMP1* was significantly increased (*p* < 0.05), and *TIMP2* was clearly expressed, accompanying the restrained degradation phenotype observed at day 1.

### 2.2. Discussion

Cutaneous wound repair outcomes result from time-dependent interactions among immune cells, fibroblasts, and the ECM, rather than isolated compartmental actions. Yet, many in vitro approaches still rely on 2D, high-stiffness substrates that distort mechanosensing and lack the spatial architecture and feedback typical of in vivo wounds [8,9]. By embedding NHDFs and THP-1 cells in a skin-derived ECM hydrogel, we demonstrate that changing the interaction mode (indirect vs. direct co-culture) shifts the system toward distinct, temporally regulated repair states. One characterized by accelerated matrix loss under paracrine exchange, and another showing early matrix deposit and a decrease in fibroblast activation under direct coculture.

The spatial organization of immune cells within the wound microenvironment determines their exposure to local cytokine gradients and matrix-derived signals. This spatial regulation plays a key role in controlling the transition from inflammation to tissue repair. However, this complex spatial dimension is difficult to reproduce in conventional 2D culture systems [10]. In monoculture, THP-1 cells readily invaded the hydrogel over time. However, in direct co-culture with NHDFs, their migration was strongly restricted. Although THP-1 cells entered the hydrogel, they remained primarily near the surface even at later time points. This observation supports the notion that fibroblasts can shape the spatial distribution of immune cells within an ECM-rich 3D environment. This aligns with the concept of the ‘stromal address code’ proposed by Davidson et al. [23]. According to this model, fibroblasts are not merely structural cells but act as immune sentinels that govern the spatial distribution and retention of leukocytes within the tissue microenvironment. The exact mechanism behind this restriction remains unclear; it could involve contact-dependent signaling, local matrix densification, or short-range inhibitor gradients. Nevertheless, this phenomenon provides a physiologically relevant readout for future investigations. The model allows for exploring matrix changes, local mechanics changes, and cell–cell interactions.

The fibroblast phenotype is significantly influenced by the dimensionality and context of the matrix [15]. In the 3D extracellular matrix, there is a promotion of cytoskeletal tension, adhesion remodeling, and activation programs that are distinct from those observed in 2D cultures on stiff plastic substrates [9]. Aligned with this body of work, we noted that encapsulating NHDFs within dermal ECM hydrogel induced and increased myofibroblast activation in comparison to 2D cultures, as evidenced by increased expression of *α-SMA*. Notably, in indirect coculture monocytes did not affect the hydrogel-induced activation. Although, in the indirect co-culture system monocytes and fibroblasts were embedded in similar hydrogels, these gels could not mechanically interact, i.e., signaling was restricted in a paracrine fashion. In direct coculture, monocytes strongly inhibited fibroblast activation already at an early stage. This indicates that in this model, proximal or distal ECM-guided mechanical crosstalk between fibroblasts and monocytes impact the 3D ECM environment such that fibroblast activation is inhibited. While our in vitro findings directly show a suppression of *α-SMA* and pro-fibrotic gene expression, from a broader wound-healing perspective, this distinction may be meaningful: excessive myofibroblast activation can lead to abnormal scarring, while controlled activation supports efficient wound healing [24]. Our results suggest that immune–fibroblast interactions are related to myofibroblast activation during wound healing. The precise causality would require targeted perturbations of adhesion or juxtacrine signaling pathways, which is a limitation of this study. Furthermore, cell–cell contact restricts deeper mechanistic insights into the signaling pathways involved.

Wound repair requires a tightly regulated balance between ECM degradation and deposition; both excessive proteolysis and excessive accumulation can disrupt healing and promote chronic inflammation or fibrosis [25]. Early-phase matrix stability is particularly consequential as it establishes the structural context for subsequent cell migration and angiogenesis [7]. In canonical fibrosis, TGF-β1 couples tightly to myofibroblast differentiation and type I collagen deposition, and *TGFB1* expression often triggers *COL1A1* induction [26]. Consistent with this paradigm, NHDF monocultures in 3D hydrogels showed high levels of both *TGFB1* and *COL1A1*. The presence of THP-1 cells suppressed *TGFB1* expression. At the same time, the canonical fibrotic outputs downstream of TGF-β1 were strongly reduced in co-culture, as reflected by a marked decrease in *COL1A1* and *ACTA2*. In this context, our collagen readouts revealed a significant divergence between interaction modes. Under indirect co-culture, characterized by paracrine exchange, collagen density decreased sharply over time. In contrast, direct co-culture displayed an early phenotype consistent with delayed or restrained collagen degradation, most evident at day 1, with a convergence observed at later time points. This temporal pattern suggests that immune-fibroblast contact does not merely “increase” or “decrease” remodeling on a global scale. Instead, it may influence the timing and intensity of proteolysis. The observation of slower early collagen degradation aligns with the concept of a potential contact-associated “early preservation” window in repair logic: maintaining a provisional scaffold in the early stages may stabilize the microenvironment for subsequent regenerative events [27]. In contrast, the premature collapse of the extracellular matrix can compromise tissue organization. Although our study does not directly quantify mechanics or ultrastructure, the histology indicates that the repair methods may diverge at early time points based on whether immune-fibroblast contact is allowed.

Protease-driven remodeling in wound environments is typically governed by the balance between MMPs and their endogenous inhibitors, TIMPs, as well as by the specific collagen programs activated by fibroblasts over time [27,28]. Under our indirect co-culture conditions, NHDFs exhibited a significant induction of *MMP1*, while *TIMP1* levels remained relatively unchanged. This observation aligns with a shift toward collagenolytic potential and the marked collagen loss noted in histological analyses. In direct co-culture, the early collagen preservation via inhibition of degradation was associated with increased *TIMP1*, detectable *TIMP2*, and relatively low net *MMP14* levels. Although transcript levels do not replace activity assays, this pattern suggests that contact-enabled conditions promote tighter regulation of pericellular proteolysis during the early stages of culture. Furthermore, the ECM synthesis program exhibited notable selectivity in direct coculture. Specifically, *ACTA2, COL1A1*, and *COL3A1* were significantly downregulated, while *COL4A1* and *FN1* were relatively preserved and returned to control-like levels later. This suggests that the coculture system targets the fibrotic ECM signature. However, it does not deplete essential components, such as those found in the basement membrane or provisional matrix. This selectivity may be advantageous in repair environments because it indicates avoidance of excessive deposition of scar-like fibrotic collagen while preserving matrix components that support tissue architecture and remodeling.

In addition to immune-mediated control of ECM remodeling, our data indicate that the ECM microenvironment and resident fibroblasts also direct monocyte phenotype in a reciprocal, context-dependent manner. Rather than following a simple M1/M2 pattern commonly described in 2D systems, THP-1 cells displayed a time-dependent adaptation consistent with niche instruction. Compared with 2D culture, the skin-derived ECM hydrogel was sufficient to induce upregulation of *CD209*, which was negligible in (stiff) 2D. This suggests that the decellularized matrix retains bioactive cues that guide incoming monocytes toward a tissue-adapted identity, even without stromal cell signals. Coculture with fibroblasts then further refined this phenotype in two complementary ways. First, direct co-culture broadly reduced macrophage surface identity. General lineage markers (*CD68*, *CD11B*) were significantly lower than in THP-1 monoculture. Canonical M2-associated markers (*CD163*, *CD206*) were also reduced. Together, these results suggest that fibroblasts limit the buildup of non-specific activation features. This restraint may help prevent persistent inflammation and excessive pro-fibrotic shift. Second, fibroblasts promoted a selective change over time. *CD209* was already detectable in the hydrogel condition. However, in direct co-culture, *CD209* increased markedly from Day 1 to Day 5 (*p* < 0.001). *CD86* also rose over time in this group. This temporal pattern was not observed in THP-1 cells cultured alone. Together, the emergence of a *CD209*-high, *CD86*-positive signature within an overall restrained marker background supports the idea that fibroblasts guide monocytes toward a more regulated state. In the context of wound repair, such *CD209*-positive phenotypes are often linked to resolution and tissue homeostasis, suggesting that immune–stromal crosstalk over time favors generation of a specialized population [29].

Angiogenesis characterizes the proliferative phase of wound healing and is controlled by the coordinated production of immune and stromal mediators, such as VEGF and FGF family members [16,18]. This crosstalk is critical because macrophages act as “chaperones” for endothelial sprouting through specialized signaling loops with fibroblasts [30]. Additionally, the composition and remodeling of the ECM affect endothelial sprouting, stability, and lumen formation. This highlights the intrinsic connection between angiogenesis and the matrix microenvironment [20]. We observed that the co-culture media significantly improved endothelial tube formation compared to baseline conditions. This suggests that the interaction between immune cells and fibroblasts alters the microenvironment and affects endothelial organization. At the transcriptional level, the expression of angiogenic factors differed depending on time and cell type. First, *VEGFA* was highly expressed in immune cells, and then *VEGFA* and *FGF2* were upregulated in the NHDFs at a later stage. During the inflammatory profile also changed dynamically. The typical pro-inflammatory signal decreased gradually, and mediators involved in later-stage signaling remain stable or increased. Although no direct causality has been identified, these changes suggest that there exists a more general regulatory mechanism. Specifically, they suggest that the co-culture system integrates inflammation control with the ability to promote blood vessel growth in skin.

Decellularized ECM hydrogels are increasingly preferred because they provide a tissue-relevant biochemistry [12,13]. However, capturing the complexity of tissue repair requires more than mixing cell types. We improve the current approach by simulating active recruitment of immune cells into a fibroblast-laden ECM scaffold. Recent immunomodulatory biomaterials show that recreating active recruitment of immune cells is essential for changing the outcome from fibrosis to regeneration [31]. One advantage of our design is the possibility of further dissecting the recruitment process by analyzing direct contact and indirect paracrine signaling separately. Comparing these modes, we demonstrated that soluble factors alone can not reproduce full immune-stromal interaction. Physical contact in recruitment was more important for controlling fibroblast activation and maintaining ECM integrity.

We acknowledge several limitations that indicate clear future directions. First, we acknowledge the limitation of relying on the immortalized THP-1 cell line. Although THP-1 cells provide a consistent and reproducible model for our 3D platform, they cannot fully reflect the complexity of primary human immune cells. Compared to primary human monocytes, THP-1 cells show biological differences, including lower CD14 expression and a weaker response to LPS. Therefore, care should be taken when interpreting these findings in the context of wound healing. To strengthen the clinical relevance of this model, future studies should confirm the main immune–stromal interactions observed here using primary human monocytes or macrophages. Second, our analysis of tissue remodeling relied primarily on gene-expression data. These results should be confirmed by measuring actual protein levels and enzymatic activity to establish functional control. Third, the concept of ‘direct contact’ is intricate and likely involves multiple mechanisms, including adhesion and short-range signaling. The study’s direct co-culture system at the stromal boundary involves initial cell seeding through mild centrifugation to prevent cell loss and synchronize matrix attachment. However, this method lacks the active chemokine-driven endothelial extravasation observed in vivo. Further research is necessary to identify the specific pathways driving these interactions. Finally, incorporating physical measurements—such as stiffness and fiber alignment—would enhance our understanding of how cellular behavior directly influences the structure of the ECM

## 3. Conclusions

We have developed a biomimetic skin-derived ECM hydrogel model based on immune cell infiltration into the wound. Unlike static co-culture systems, this model captures the recruitment stage of wound healing and allows for monocytes to enter fibroblast-containing matrix and interact with resident cells over time. Using this physiologically relevant setup, we show that immune cell infiltration is not a response to inflammation; rather, the physical entry of monocytes into the dermal matrix—which was restricted to a depth of 8.92 ± 2.27 μm by fibroblasts compared to 121.1 ± 15.9 μm in monoculture at day 5—is associated with the mitigation of rapid tissue remodeling. Early immune cell recruitment is associated with reduced collagen fiber degradation, maintaining a high collagen intensity ratio of 3.46 ± 0.33 compared to the rapid drop to 2.02 ± 0.29 observed in fibroblast monocultures at day 1. This shifts the proteolytic balance by increasing *TIMPs* expression and suppressing pro-fibrotic gene programs in fibroblasts. Importantly, this 3D model reproduces two requirements of wound healing. On the one hand, hydrogel-induced activation limits myofibroblast activity, reducing early *α-SMA* expression from 79.1 ± 4.9% to 54.3 ± 5.7%, thus preventing fibrotic overactivation. On the other hand, the secretory environment, supported by a >2.5-fold amplification of *VEGFA* in co-cultured fibroblasts, promotes endothelial tube formation and supports angiogenesis. In summary, our model provides a physiologically relevant bridge between simplified 2D cultures and the complex in vivo wound niche. By including immune cell infiltration as an active process, this platform provides a useful tool for studying how inflammation, matrix stability, and fibrosis are balanced during early tissue repair.

## 4. Materials and Methods

### 4.1. Cell Culture

NHDFs and the human monocytic cell line THP-1 were obtained from ATCC. Human HUVECs were obtained from the endothelial cell culture facility at UMCG. NHDFs were maintained in DMEM (Lonza, Basel, Switzerland) supplemented with 10% FBS, 1% penicillin-streptomycin, and 1% L-glutamine, and passages 6–10 were used in the experiments. THP-1 cells were cultured in RPMI 1640 (Lonza) containing 10% FBS, 1% penicillin-streptomycin, and 1% L-glutamine. HUVECs were grown in endothelial cell medium composed of RPMI 1640 supplemented with 20% heat-inactivated FBS, 1% penicillin-streptomycin, 1% L-glutamine, 5 U/mL heparin (LEO Laboratories Limited, Ballerup, Denmark), and 20 µg/mL endothelial growth factor prepared from home-made bovine brain extract. HUVECs from passages 4–6 were used. Unless stated otherwise, cells were cultured at 37 °C in a humidified incubator with 5% CO_2_.

### 4.2. Preparation of Skin-Derived ECM Hydrogels

Skin-derived ECM hydrogels were prepared following previously established protocols [16]. Importantly, to ensure complete decellularization and minimize batch-to-batch variability, the residual DNA content of the resulting dried ECM was strictly controlled to be less than 50 ng/mg dry weight. As comprehensively characterized in our previous reports [16,20,32], these hydrogels feature a highly interconnected, porous nanofibrous network and a soft mechanical stiffness that closely mimics the physiological properties of native human dermis, thereby providing a highly biomimetic rationale for using this platform to study cell infiltration. To ensure optimal reproducibility, the specific decellularization and gelation procedures are detailed below in sequential phases:

Fresh porcine skin, sourced from a local abattoir (Kroon Vlees, Groningen, The Netherlands), was sectioned into approximately 1 cm^3^ fragments. These pieces were suspended in chilled Dulbecco’s phosphate-buffered saline (DPBS; Lonza, Walkersville, MD, USA) and blended into a uniform paste using a commercial food processor. The suspension was subsequently subjected to ultrasonic homogenization Sigma-Aldrich, Zwijndrecht, The Netherlands) at maximum amplitude for 60 s. After centrifugation and two successive DPBS washings, the tissue paste was treated chemically and enzymatically to remove cell-specific components. The paste was first agitated in 0.05% trypsin/DPBS for 4 h at 37 °C, then washed for 3 h in Milli-Q^®^ water at 37 °C, and finally incubated overnight at 37 °C in 6 M NaCl. The next overnight incubation at 37 °C involved exposure to 1% SDS and 1% Triton X-100 (Sigma-Aldrich), followed by 1% sodium deoxycholate and 30 g/mL DNase (Roche Diagnostics, Mannheim, Germany) in Milli-Q^®^ water with 1.3 mM MgSO_4_ and 2 mM CaCl_2_. The intermediate product was rinsed three times with Milli-Q^®^ water between each chemical treatment. The crude decellularized matrix was washed six times with DPBS (1 h per wash cycle) under continuous stirring. After pelleting (3000× *g*) centrifugation, the matrix was sterilized overnight at room temperature with 70% ethanol. The sterilized ECM was snap-frozen in liquid nitrogen, fully lyophilized, and pulverized into fine particulate powder using an Ultra-Turrax homogenizer (IKA, Staufen, Germany). Hydrogel formulation and Neutralization: The lyophilized ECM powder was suspended at 20 mg/mL and digested with porcine pepsin (1 mg/mL) in 0.01 M HCl. The digest was kept on ice for 24 h to achieve physiological pH (7.4) and isotonicity by mixing in 1/10 volume of 1 M NaOH and 1/10 volume of 10 DPBS. The neutral ECM pre-gel was stored at 4 °C until use to prevent thermal crosslinking.

### 4.3. Establishment of 3D Coculture Models

**Figure 10:**
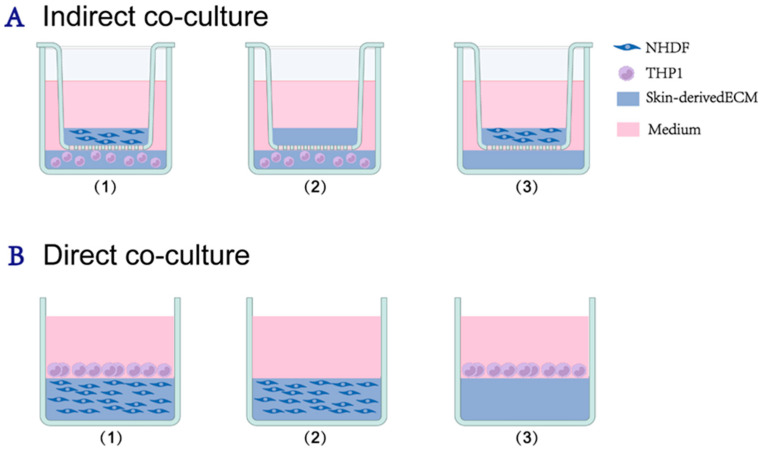
Experimental design and grouping. (**A**) Indirect co-culture system: This setup evaluated soluble factor exchange. The experimental groups were designated as follows: (1) co-culture group, consisting of skin-derived hydrogels embedded with NHDFs in the upper insert and THP-1-encapsulated hydrogels in the lower chamber; (2) THP-1 monoculture control, containing only THP-1 cells in the hydrogel in the lower chamber; and (3) NHDF monoculture control, containing only NHDFs in the hydrogel in the upper insert. On day 1, conditioned medium (CM) was harvested for HUVEC tube formation assays. (**B**) Direct co-culture system: NHDF-embedded hydrogels were cast and gelated in 48-well plates. Subsequently, THP-1 cell suspensions were added onto the solidified gel surfaces, followed by centrifugation at 112× *g* for 5 min to facilitate the physical adhesion of monocytes to the hydrogel interface. Experimental grouping: (1) THP-1—NHDF co-culture group; (2) NHDF monoculture control, consisting of NHDF-embedded hydrogels without immune cells; and (3) THP-1 monoculture control, where THP-1 cells are seeded on top of cell-free hydrogels. Created with BioRender.com.

To study the roles of soluble signaling versus physical recruitment, two distinct coculture configurations were established (Figure 10). RPMI 1640 supplemented with 10% FBS, 1% penicillin-streptomycin, and 1% L-glutamine was used as the culture medium throughout.

#### 4.3.1. Indirect Coculture System (Paracrine Model)

A 24-well Transwell system (0.4 μm pore size, Corning) was used to permit soluble factor exchange without cell contact. NHDFs (1.5 × 10^6^ cells/mL) were encapsulated in 100 µL ECM hydrogel and seeded into the upper insert. THP1 cells (5 × 10^6^ cells/mL) were encapsulated in 400 µL hydrogel and seeded into the lower chamber. Controls included NHDF-embedded hydrogels (upper) and THP1-embedded hydrogels (lower) cultured alone. Then, the samples were placed in a 37 °C incubator with 5% CO_2_ for 40 min to allow gelation, after which 200 µL of culture medium was added to the upper chamber and 1.5 mL to the lower chamber. All samples were incubated at 37 °C in a 5% CO_2_ incubator for 5 days, with the culture medium refreshed every 2 days.

To facilitate clarity, a combined naming convention was adopted for the indirect system, where the prefix and suffix denote the contents of the upper chamber and the lower chamber, respectively. Specifically: NHDF-ECM: NHDF-embedded hydrogel in the upper chamber and cell-free ECM hydrogel in the lower chamber. ECM-THP-1: Cell-free ECM hydrogel in the upper chamber and THP-1-embedded hydrogel in the lower chamber. NHDF-THP-1: NHDF-embedded hydrogel in the upper chamber and THP-1-embedded hydrogel in the lower chamber.

#### 4.3.2. Direct Coculture System (Infiltration Model)

To simulate the active recruitment of monocytes into the stroma, a sequential layering method was employed.

- Experimental group (co-culture):

Matrix formation: NHDFs (1.5 × 10^6^ cells/mL) were encapsulated in 200 µL of neutralized ECM solution, in 48-well plates, and allowed to gelate at 37 °C for 40 min.

Infiltration: A 400 µL suspension of THP-1 cells (1.0 × 10^6^ cells/mL) was added directly on top of the solidified NHDF-embedded hydrogel.

Physical recruitment & gradient induction: To mimic the recruitment process, the plates were centrifuged at 112× *g* for 5 min. Notably, this centrifugation step served a dual function: beyond facilitating immediate physical adhesion of THP-1 cells to the hydrogel interface, it induced vertical structural stratification within the matrix. This created a distinct upper low-density collagen region and a lower high-density collagen region. This architectural gradient was subsequently utilized to distinguish depth-dependent remodeling dynamics in the ratiometric collagen analysis.

- Control groups:

NHDF monoculture control: NHDFs (1.5 × 10^6^ cells/mL) were encapsulated in 200 µL of ECM hydrogel. After polymerization, 400 µL of cell-free culture medium was added on top, followed by centrifugation (112× *g*, 5 min). This ensured that the monoculture control underwent the same mechanical compression and density stratification as the co-culture group, providing a structurally comparable baseline.

THP-1 monoculture control: Cell-free ECM hydrogels (200 µL) were prepared and gelated. Subsequently, a 400 µL suspension of THP-1 cells (1.0 × 106 cells/mL) was seeded onto the surface of the cell-free gel, followed by centrifugation (112× *g*, 5 min). Cultures were run up to 5 days.

For both co-culture systems described above, Day 0 was defined as the time point 4 h post-gelation.

### 4.4. Histology and Immunohistochemistry (IHC)

At days 0, 1, and 5, hydrogel constructs were fixed with 4% paraformaldehyde for 20 min. To minimize gel deformation during processing, samples were first embedded in 2% agarose (Invitrogen, Carlsbad, CA, USA) before dehydration. After paraffin embedding, 4 μm sections were prepared for histological analysis.

Immunohistochemistry (IHC): Paraffin sections were deparaffinized and rehydrated prior to antigen retrieval. For α-SMA staining, retrieval was carried out in 0.1 M Tris/HCl buffer (pH 9.0), whereas Tris/EDTA buffer (pH 9.0) was used for CD45 staining. After the blocking step, sections were incubated overnight at 4 °C with primary antibodies against CD45 ((1:300, Abcam, Cambridge, UK, ab10558)) to identify THP-1 cells and α-SMA (1:200, Abcam, ab7817) to detect activated fibroblasts. The corresponding secondary antibodies were polyclonal rabbit anti-mouse for α-SMA (1:100, Dako, Glostrup, Denmark) and polyclonal swine anti-rabbit for CD45 (1:100, Dako, Glostrup, Denmark). Signal development was achieved with DAB substrate (Sigma-Aldrich, St. Louis, MO, USA), and nuclei were counterstained using hematoxylin.

Picrosirius red (PSR) staining: Collagen fiber density was evaluated by staining sections with 0.1% picrosirius red (Sigma-Aldrich) for 1 h. After staining, sections were rinsed twice in 0.5% acetic acid, dehydrated through three changes in absolute ethanol, cleared in xylene, and mounted with a resin-based mounting medium.

All stained slides were scanned at 40× magnification using a whole-slide scanner (Hamamatsu Photonics, Hamamatsu City, Japan). The digital images were subsequently used for both qualitative assessment and quantitative analysis.

### 4.5. RNA Isolation and Quantitative Real-Time PCR (RT-qPCR)

Total RNA was extracted using TRIzol™ Reagent (Thermo Fisher Scientific, Waltham, MA, USA). For the direct co-culture samples, whole hydrogels were homogenized to evaluate the combined gene expression profile of both interacting cell types within the same microenvironment. Reverse transcription was carried out with RevertAid Reverse Transcriptase (Thermo Scientific, Waltham, MA, USA). qPCR was performed using FastStart Universal SYBR Green Master and the primer sets listed in Table 1. Reactions were run in 384-well PCR plates (Thermo Scientific, USA) on a ViiA 7 Real-Time PCR System (Applied Biosystems, Carlsbad, CA, USA). Relative expression levels were determined from ΔCt values normalized to *GAPDH*.

### 4.6. HUVEC Tube Formation Assay

Conditioned media (CM) were harvested from the indirect co-culture system and controls after 24 h (Day 1). HUVECs (8.0 × 10^5^ cells/mL) were seeded onto Matrigel-coated Nunc™ MiniTrays with Nunclon™ Delta surface (60-well, 163118, Thermo Fisher Scientific), and incubated with the CM for 2 h. Tube formation was imaged using bright-field of an inverted fluorescence microscope (EVOS, Thermo Fisher) at 10× magnification. The network complexity, specifically the number of junctions and the number of segments, was quantified using the Angiogenesis Analyzer plugin in Fiji [33].

### 4.7. Quantitative Image Analysis

All image quantification was performed using Fiji (version 1.53t) [34].

THP-1 migration depth: In the direct co-culture system, vertical migration was quantified on CD45-stained sections. For each field, the five THP-1 cells located farthest from the hydrogel surface were identified. The perpendicular distance from the hydrogel surface to the center of each cell was measured, and the mean value was calculated as the migration depth (Figure 2).

Fibroblast activation: The percentage of α-SMA-positive cells was calculated as: (Number of DAB-positive NHDFs/Total number of hematoxylin-stained NHDFs) × 100.

Collagen Remodeling Analysis: Indirect co-culture: The mean gray value of collagen fibers was measured in random regions of interest (ROIs). Background intensity was subtracted. Direct co-culture (Ratiometric Analysis): To assess depth-dependent degradation, collagen intensity was measured in the upper region and the lower region. The intensity ratio was calculated as shown in the equation below. A ratio > 1 indicates preservation of the ECM.

$$Ratio=\frac{Mean\;Intensity\;(Lower)}{Mean\;Intensity\;(Upper)}$$

### 4.8. Statistical Analysis

Data were presented as Mean ± Standard Error of the Mean (SEM). Statistical analyses were performed using GraphPad Prism (version 10.0.0 for Windows, GraphPad Software, Boston, MA, USA). The exact number of independent experiments and biological/technical replicates (*n*) for each specific assay is detailed in the respective figure legends. Multiple comparisons were analyzed using one-way ANOVA or two-way ANOVA (to assess Time × Condition interactions), followed by Tukey’s post hoc test. A *p*-value < 0.05 was considered statistically significant.

## Figures and Tables

**Table 1:** Human primer sequences used for RT-qPCR.

Target	Forward Primer (5′ → 3′)	Reverse Primer (5′ → 3′)
*GAPDH*	AGCCACATCGCTCAGACAC	GCCCAATACGACCAAATCC
*CD68*	CAGGGAATGACTGTCCTCACA	TCCAGTGCTCTCTGTAACCG
*CD11b*	CAGGTTCTGGCTCCTTCCAG	GATCCCTGAAGCTGGACCAC
*CD86*	GCAGAAGCAGCCAAAATGGA	AGCTCACTCAGGCTTTGGTT
*CD163*	GCAAGTGGCCTCTGTAATCT	AGCACTTTCTTCTGGAATGG
*CD206*	TTCCTTTGGACGGATGGACG	CCTCGTTTACTGTCGCAGGT
*CD209*	TCAAAAGTGCTGAGGAGCAG	TGGGCTCTCCTCTGTTCCAA
*TNFA*	CAGCCTCTTCTCCTTCCTGAT	GCCAGAGGGCTGATTAGAGA
*IL10*	ACCTCCGCCAATCTCTCACT	CTCAGACAAGGCTTGGCAAC
*IL1B*	AAGCTGGAATTTGAGTCTGC	ACACAAATTGCATGGTGAAG
*IL6*	AGCTCAATAAGAAGGGGCCTA	TGAGAAACCCTGGCTTAAGTAGA
*MMP1*	GCTAACCTTTGATGCTATAACTACGA	TTTGTGCGCATGTAGAATCTG
*MMP2*	GTTCCCCTTCTTGTTCAATG	CTTGCCATCCTTCTCAAAGT
*MMP9*	GACGATGACGAGTTGTGGT	GAAGATGAAGGGGAAGTGG
*MMP14*	GGGTGAGGAATAACCAAGTG	CTTCCTCTCGTAGGCAGTGT
*TIMP1*	CCAGCGTTATGAGATCAAGA	AGTATCCGCAGACACTCTCC
*TIMP2*	GAAGAGCCTGAACCACAGGT	CGGGGAGGAGATGTAGCAC
*TGFB1*	ACTACTACGCCAAGGAGGTCAC	TGCTTGAACTTGTCATAGATTTCG
*VEGFA*	CCTGAAATGAAGGAAGAGGA	AAATAAAATGGCGAATCCAA
*FGF2*	CTGTACCCATACAGCAGCAG	CGCCTAAAGCCATATTCATT
*COL1A1*	GGGATTCCCTGGACCTAAAG	GGAACACCTCGCTCTCCA
*COL3A1*	CTGGACCCCAGGGTCTTC	CATCTGATCCAGGGTTTCCA
*COL4A1*	CAGCAACGAACCCTAGAAAT	CAATGAAGCAGGGTGTGTTA

## Data Availability

The data supporting the findings of this study are available within the article.

## References

[1] Singhvi G., Manchanda P., Rapalli V.K., Dubey S.K., Gupta G., Dua K. (2018). MicroRNAs as biological regulators in skin disorders. Biomed. Pharmacother..

[2] Kobayashi T., Nagao K. (2019). “Deepening” Insight on Skin Aging and Anti-microbial Immunity. Cell Metab..

[3] Lin X., Zhang X., Wang Y., Chen W., Zhu Z., Wang S. (2025). Hydrogels and hydrogel-based drug delivery systems for promoting refractory wound healing: Applications and prospects. Int. J. Biol. Macromol..

[4] del Fresno C., Sancho D. (2020). Clec2d Joins the Cell Death Sensor Ranks. Immunity.

[5] Lu D., Jiao X., Jiang W., Yang L., Gong Q., Wang X., Wei M., Gong S. (2023). Mesenchymal stem cells influence monocyte/macrophage phenotype: Regulatory mode and potential clinical applications. Biomed. Pharmacother..

[6] Banjac I., Maimets M., Jensen K.B. (2023). Maintenance of high-turnover tissues during and beyond homeostasis. Cell Stem Cell.

[7] Sylakowski K., Bradshaw A., Wells A. (2020). Mesenchymal Stem Cell/Multipotent Stromal Cell Augmentation of Wound Healing Lessons from the Physiology of Matrix and Hypoxia Support. Am. J. Pathol..

[8] Vermeulen S., Birgani Z.T., Habibovic P. (2022). Biomaterial-induced pathway modulation for bone regeneration. Biomaterials.

[9] Zhao F., Zhang M., Nizamoglu M., Kaper H.J., Brouwer L.A., Borghuis T., Burgess J.K., Harmsen M.C., Sharma P.K. (2024). Fibroblast alignment and matrix remodeling induced by a stiffness gradient in a skin-derived extracellular matrix hydrogel. Acta Biomater..

[10] Grist S.M., Nasseri S.S., Laplatine L., Schmok J.C., Yao D., Hua J., Chrostowski L., Cheung K.C. (2019). Long-term monitoring in a microfluidic system to study tumour spheroid response to chronic and cycling hypoxia. Sci. Rep..

[11] Denchai A., Tartarini D., Mele E. (2018). Cellular Response to Surface Morphology: Electrospinning and Computational Modeling. Front. Bioeng. Biotechnol..

[12] Crapo P.M., Gilbert T.W., Badylak S.F. (2011). An overview of tissue and whole organ decellularization processes. Biomaterials.

[13] Watt F.M., Fujiwara H. (2011). Cell-Extracellular Matrix Interactions in Normal and Diseased Skin. Cold Spring Harb. Perspect. Biol..

[14] Grinnell F. (2003). Fibroblast biology in three-dimensional collagen matrices. Trends Cell Biol..

[15] de Menezes J., Saraiva E., da Rocha-Azevedo B. (2016). The site of the bite: Leishmania interaction with macrophages, neutrophils and the extracellular matrix in the dermis. Parasites Vectors.

[16] Zhang M., Zhao F., Zhang X., Brouwer L.A., Burgess J.K., Harmsen M.C. (2023). Fibroblasts alter the physical properties of dermal ECM-derived hydrogels to create a pro-angiogenic microenvironment. Mater. Today Bio.

[17] Tonnesen M., Feng X., Clark R. (2000). Angiogenesis in wound healing. J. Investig. Dermatol. Symp. Proc..

[18] Mao X., Xu J., Wang W., Liang C., Hua J., Liu J., Zhang B., Meng Q., Yu X., Shi S. (2021). Crosstalk between cancer-associated fibroblasts and immune cells in the tumor microenvironment: New findings and future perspectives. Mol. Cancer.

[19] Ribatti D. (2024). Different subpopulations of macrophages, neutrophils, mast cells, and fibroblasts are involved in the control of tumor angiogenesis. Front. Med..

[20] Zhang M., Zhao F., Zhu Y., Brouwer L.A., Van der Veen H., Burgess J.K., Harmsen M.C. (2024). Physical Properties and Biochemical Composition of Extracellular Matrix-Derived Hydrogels Dictate Vascularization Potential in an Organ-Dependent Fashion. ACS Appl. Mater. Interfaces.

[21] Spiekman M., Francia D.L., Mossel D.M., Brouwer L.A., Diercks G.F.H., Vermeulen K.M., Folkertsma M., Ghods M., Kzhyshkowska J., Klüter H. (2021). Autologous Lipofilling Improves Clinical Outcome in Patients with Symptomatic Dermal Scars Through Induction of a Pro-Regenerative Immune Response. Aesthetic Surg. J..

[22] Zhang X., Schipper J.A.M., Schepers R.H., Jansma J., Spijkervet F.K.L., Harmsen M.C. (2024). A Versatile Skin-Derived Extracellular Matrix Hydrogel-Based Platform to Investigate the Function of a Mechanically Isolated Adipose Tissue Stromal Vascular Fraction. Biomolecules.

[23] Davidson S., Coles M., Thomas T., Kollias G., Ludewig B., Turley S., Brenner M., Buckley C.D. (2021). Fibroblasts as immune regulators in infection, inflammation and cancer. Nat. Rev. Immunol..

[24] Abdalla M.M.I., Mohanraj J., Somanath S.D. (2023). Adiponectin as a therapeutic target for diabetic foot ulcer. World J. Diabetes.

[25] Horowitz J.C., Ajayi I.O., Kulasekaran P., Rogers D.S., White J.B., Townsend S.K., White E.S., Nho R.S., Higgins P.D., Huang S.K. (2012). Survivin expression induced by endothelin-1 promotes myofibroblast resistance to apoptosis. Int. J. Biochem. Cell Biol..

[26] Hinz B. (2007). Formation and Function of the Myofibroblast during Tissue Repair. J. Investig. Dermatol..

[27] Daley W.P., Peters S.B., Larsen M. (2008). Extracellular matrix dynamics in development and regenerative medicine. J. Cell Sci..

[28] Gill S.E., Parks W.C. (2008). Metalloproteinases and their inhibitors: Regulators of wound healing. Int. J. Biochem. Cell Biol..

[29] Lugo-Villarino G., Troegeler A., Balboa L., Lastrucci C., Duval C., Mercier I., Bénard A., Capilla F., Al Saati T., Poincloux R. (2018). The C-Type Lectin Receptor DC-SIGN Has an Anti-Inflammatory Role in Human M(IL-4) Macrophages in Response to Mycobacterium tuberculosis. Front. Immunol..

[30] Tay H., Cheyne C.D., Demeyere K., De Craene J., De Bels L., Meyer E., Zijlstra A., De Spiegelaere W. (2021). Depletion of Embryonic Macrophages Leads to a Reduction in Angiogenesis in the Ex Ovo Chick Chorioallantoic Membrane Assay. Cells.

[31] Sadtler K., Singh A., Wolf M.T., Wang X., Pardoll D.M., Elisseeff J.H. (2016). Design, clinical translation and immunological response of biomaterials in regenerative medicine. Nat. Rev. Mater..

[32] Vriend L., van Dongen J.A., Sinkunas V., Brouwer L.A., Buikema H.J., Moreira L.F., Gemperli R., Bongiovanni L., de Bruin A., van der Lei B. (2022). Limited Efficacy of Adipose Stromal Cell Secretome-Loaded Skin-Derived Hydrogels to Augment Skin Flap Regeneration in Rats. Stem Cells Dev..

[33] Carpentier G., Berndt S., Ferratge S., Rasband W., Cuendet M., Uzan G., Albanese P. (2020). Angiogenesis Analyzer for ImageJ—A comparative morphometric analysis of “Endothelial Tube Formation Assay” and “Fibrin Bead Assay”. Sci. Rep..

[34] Schindelin J., Arganda-Carreras I., Frise E., Kaynig V., Longair M., Pietzsch T., Preibisch S., Rueden C., Saalfeld S., Schmid B. (2012). Fiji: An open-source platform for biological-image analysis. Nat. Methods.

